# Carboxylated ε-Poly-L-Lysine Supplementation of the Freezing Extender Improves the Post-Thawing Boar Sperm Quality

**DOI:** 10.3390/ani12131726

**Published:** 2022-07-04

**Authors:** Weijing Zhang, Yajing Li, Zhendong Zhu

**Affiliations:** College of Animal Science and Technology, Qingdao Agricultural University, Qingdao 266109, China; zhangweijing202103@163.com (W.Z.); lyj2019521@163.com (Y.L.)

**Keywords:** boar sperm, cryopreservation, CPLL, quality

## Abstract

**Simple Summary:**

Frozen boar sperm is used on a very limited scale in pig artificial insemination owing to the low quality of post-thaw sperm. Cryoprotectant is usually used in boar sperm freezing extender, which is important for improving the post-thaw sperm quality. The carboxylated ε-poly-L-lysine, an efficient and non-toxic cryoprotectant, has been used as a food and cell preservative, as well as for tissue engineering and drug delivery in the biomedical applications. However, whether addition of carboxylated ε-poly-L-lysine to the freezing medium improves the post-thaw boar sperm quality or not is unknown. In this study, the addition of 0.25% carboxylated ε-poly-L-lysine to the freezing medium significantly improved the post-thaw boar sperm quality by protecting sperm mitochondrial function and antioxidant defense system.

**Abstract:**

Frozen boar sperm is used on a minimal scale in consequence of the cryo-injuries induced by biochemical and physical modifications during the freezing and thawing processes. The present study investigates whether the addition of carboxylated ε-poly-L-lysine (CPLL) to the freezing medium could improve post-thaw boar sperm quality or not. Boar sperm was diluted with freezing medium contained different doses of carboxylated ε-poly-L-lysine (0, 0.125%, 0.25%, 0.5%, and 1%; *v/v*). The motility patterns, membrane integrity, acrosome integrity, mitochondrial membrane potential, NADH-CoQ activity, ATP level, malondialdehyde (MDA) level, and antioxidant defense system, as well as apoptosis in post-thaw boar sperm, were measured. It was observed that 0.25% CPLL treatment significantly improved the post-thaw boar sperm total motility, progressive motility, straight-linear velocity (VSL), curvilinear velocity (VCL), average path velocity (VAP), linearity (LIN), straightness (STR), membrane integrity, and acrosome integrity. Interestingly, the addition of CPLL also significantly increased the post-thaw sperm mitochondrial membrane potential, NADH-CoQ activity, and ATP level. Moreover, post-thaw boar sperm catalase (CAT) activity, glutathione peroxidase (GPx) activity, and superoxide dismutase (SOD) activity were increased with the addition of CPLL from 0.125% to 0.5% concentration levels. Furthermore, reduction of post-thaw sperm MDA level and apoptosis in 0.25% CPLL treatment was also observed. Those observations suggested that the addition of 0.25% CPLL to the freezing medium increased post-thaw boar sperm quality by protecting sperm mitochondrial function and antioxidant defense system. These findings provided novel insights that CPLL can be used as an efficient cryoprotectant to improve the post-thaw boar sperm quality during cryopreservation.

## 1. Introduction

Artificial insemination (AI) is widely used in pig production around the world [1,2]. The outcome of AI not only depends on the insemination procedure but is also largely affected by the semen quality [3]. In pigs, the liquid-stored semen is mostly used for intracervical insemination, while less than 1% of AI is performed using frozen-thaw sperm [1,4]. The outcomes of birth rate and little size in AI carried out using frozen-thaw sperm are much lower compared to when liquid-stored semen was used [4]. In addition, the lifespan of frozen-thaw boar sperm in the gilts’ reproductive tract was shorter when compared to the liquid-stored sperm in vivo [5]. Thus, developing a novel method to improve the post-thaw boar sperm quality will contribute to the application of frozen-thaw sperm in pig AI.

Sperm cryopreservation includes a cooling, freezing, and thawing processes. Due to the dramatic changes in sperm’s physical and chemical surroundings in sperm cryopreservation procedures [3,6], sperm suffers irreversible damages, such as a decrease in DNA integrity [6], interruption of membrane permeability and transition [7], and impaired cellular calcium homeostasis [8]. In a bid to improve post-thaw boar sperm quality, several studies have focused on the modification of boar sperm freezing and thawing procedures, such as the addition of antioxidants [9], BSA [10], EGTA [11], or the addition of anti-freeze proteins [12] to the freezing medium, as well as supplementation of seminal plasma to the thawing medium [13]. In our previous study [14], it was also observed that a reduction of the glucose level in the pre-treatment medium could increase the post-thaw boar sperm quality by inactivating the glycolysis pathway to protect sperm from glucose-toxic damage.

Cryoprotectants, known as cryoprotective agents (CPAs), are used to protect the cells from damage induced by the cooling, freezing, and thawing processes during cryopreservation [15]. It is generally accepted that the presence of CPAs both in the extra and intracellular medium can increase viscosity and considerably reduce nucleation and aggregation of crystals [15,16], thereby decreasing the sperm cryo-damage. Recently, carboxylated ε-poly-L-lysine (CPLL) has been utilized, a bioinspired biocompatible material that has been used as a food and cell preservative as well as for tissue engineering and drug delivery in the biomedical applications because of its reduced toxicity and cryoprotective properties [17,18]. In addition, CPLL has also been reported for use as an additional CPA in the cryopreservation of numerous cell types such as the red blood cells [19], natural killer cells [17], stem cells [20], and bovine somatic cells [21]. Furthermore, CPLL showed a higher cryoprotective property effect on pig embryos [22] and Nili-Ravi buffalo bull sperm [23]. However, the cryoprotective effects of CPLL on boar sperm have not been investigated in the boar sperm cryopreserved regimes. Additionally, the exact mechanism of CPLL on sperm cryopreservation is unknown. Therefore, the present study aims to explore whether the addition of CPLL to the freezing medium will improve the post-thaw boar sperm quality.

## 2. Materials and Methods

### 2.1. Chemicals and Extenders

All chemicals and reagents were purchased from Sigma-Aldrich China unless specified otherwise.

The pre-treatment solution was composed of 30.6 mM D-glucose, 122.4 mM lactose, 26.7 mM trisodium citrate, 11.9 mM sodium hydrogen carbonate, 15.1 mM citric acid, 6.3 mM EDTA-2Na, 46.6 mM Tris, 1000 IU/mL penicillin G sodium salt (Solarbio, Beijing, China), 100 μg/mL polymyxin B, and 1 mg/mL streptomycin sulfate (Solarbio), as described by Zhu et al. (2022) [14]. According to our previous studies [9,14], freezing extender with some modifications (mNSF1) was used in the present study. The mNSF1 consisted of 80% (*v/v*) of 310 mM lactose monohydrate, 20% (*v/v*) of egg yolk and 1000 IU/mL penicillin G sodium salt, 100 μg/mL polymyxin B, and 1 mg/mL streptomycin sulfate. The second dilution (mNSF2) contained 1.5% (*v/v*, final concentrations: 0.75%) Orvus Es Paste (OEP) (Miyazaki Chemical Sales, Ltd., Tokyo, Japan), 2% glycerol (*v/v*, final concentrations: 1%), and different concentrations of CPLL (*v/v*, final concentrations: 0, 0.25%, 0.5%, 0.75%, 1%). CPLL solution preparation: Epsilon poly L-Lysine (PLL, Macklin Chemical Co., Shanghai, China) was dissolved (25%; *w/v*) in Milli-Q water, and then 1.3 g of succinic anhydride was mixed per 10 mL of PLL solution, and then heated at 50 °C for 1 h.

### 2.2. Semen Collection

In this study, five mature and fertile Duroc boars were used to collect semen for experiment. The average age of boars was 18 months, and the weight was 200 kg to 250 kg. All animals and experimental procedures were approved by the Qingdao Agriculture University Institutional Animal Care and Use Committee (QAU1121010). The boars were housed in individual pens of 8 m^2^ area with a concrete floor and fed with a commercial diet. Drinking water was available ad libitum. Gloved-hand technique was used to collect sperm-rich fraction semen in each boar once per week. Five ejaculates from each boar were used, and only samples with more than 75% of total motility and less than 15% sperm abnormalities were used in this study.

### 2.3. Semen Processing

According to Zhu et al. (2022) [14], the fresh semen was diluted with a pre-treatment solution and stocked for 2 h at 15 °C, then centrifuged with 700× *g* for 10 min to collect the sperm pellet. mNSF1was used to resuspend sperm pellet with a concentration of 2.0 × 10^9^ sperm/mL and slowly cooled to 5 °C. After cooling, the sperm suspension was divided into 5 parts and diluted with the mNSF2 (*v/v* = 1:1) containing 1% glycerol, 1.5% OEP, and various concentration levels of CPLL (0%, 0.25%, 0.5%, 0.75%, 1%). Subsequently, the diluted semen was packed into a 0.5 mL plastic straw and placed horizontally 5 cm above the surface of liquid nitrogen for 10 min. After that, the straws were plunged into liquid nitrogen for storage. The frozen straw was transferred to water at 37 °C for 20 s and diluted in 4.5 mL of pre-treatment solution.

### 2.4. Evaluation of Post-Thaw Sperm Motility

Sperm motility was assessed with computer-assisted sperm analysis (CASA) system (Integrated Semen Analysis System; Hview, Fuzhou, China). The standard parameter settings were set at 25 frames/s and the images were obtained using a negative contrast phase microscope at 100× magnification (Leica, Wetzlar, Germany) and a digital camera (Basler Vision, Ahrensburg, Germany). According to our previous study [14], 5 μL aliquot of the post-thaw semen sample was added to a prewarmed analyzer’s Makler chamber after incubation for 15 min at 37 °C, and then three fields were randomly selected for evaluation of the post-thaw sperm motility in each treatment by the CASA, more than 500 sperm were evaluated. Total motility was defined as the percentage of motile sperm moving with a path velocity >12 μm/s. Progressive motility was expressed as percentage of motile sperm moving with path velocity of 45 μm/s and in a straight line for over 80% of the time.

### 2.5. Evaluation of Sperm Membrane Integrity and Acrosome Integrity

LIVE/DEAD Sperm Viability Kit and fluorescein isothiocyanate-peanut agglutinin were used to measure sperm membrane integrity and acrosome integrity, respectively. Briefly, for membrane integrity detection, 120 μL sperm sample were incubated with 0.12 μL of 100 nM SYBR-14 working solution and 0.6 μL of 2.4 mM PI stocking solution for 10 min in the dark; meanwhile, 100 μg/mL fluorescein isothiocyanate-peanut agglutinin solution and 2.4 mM solution were incubated with sperm sample after fixed with methanol to evaluate acrosome integrity. The stained sperm was monitored and photographed with an epifluorescence microscope with a set of filters (400×) with 516 nm emission for green fluorescence and 617 nM emission for red fluorescence according to Zhu et al. (2020) [24].

### 2.6. Mitochondrial Membrane Potential

Sperm mitochondrial membrane potential was analyzed with the JC-1 Mitochondrial Membrane Potential Detection Kit (Beyotime Institue of Biotechnology, Shanghai, China) according to our previous study [25]. Briefly, sperm samples were stained with 1× JC-1 working solution for 30 min in the dark. Monomer and aggregates are the two types of JC-1stained mitochondrial plasma. The monomer emits green fluoresce while the aggregates emit red yellow–orange fluoresce over the midpiece. The stained sperm were analyzed with a flow cytometer (574/26 nm for red fluorescence, 530/30 nm for green fluorescence) (FACSCalibur, BD Biosciences). The percentage of red stained cells was considered as the population of sperm with high mitochondrial membrane potential. A total of 20,000 sperm events were analyzed.

### 2.7. Measurement of NADH-CoQ Activity

Sperm NADH-CoQ activity was measured with a commercial kit (A089-1-1, Nanjing Jiancheng Bioengineering Institute, Nanjing, China) following the manufacturer’s protocol. Briefly, the samples were lysed and centrifuged to collect the supernatant. Then, the supernatant was mixed with the assay buffer and substrates and measured at 570 nm with a microplate reader.

### 2.8. Measurement of Sperm ATP Level

Sperm ATP level was measured with an ATP Assay Kit (S0026, Beyotime Institute of Biotechnology, Shanghai, China) according to the previous study [24]. Briefly, the samples were mixed with the assay buffer and substrates, and then the luminescence was measured with a luminometer (Thermo Scientific, Palm Beach, FL, USA).

### 2.9. Measurement of Post-Thaw Sperm MDA Content

According to our previous study [9], malonaldehyde (MDA) content was measured with a commercial lipid peroxidation MDA assay kit (S0131S, Beyotime Institute of Biotechnology, Shanghai, China). Briefly, post-thaw sperm in each treatment were lysed and then centrifuged to collect the supernatant. The supernatant was mixed with the preparing reaction buffer following the manufacturer’s protocol and measured at 532 nm with a microplate reader. MDA levels were then normalized to milligram protein.

### 2.10. Evaluation of Activities of CAT, SOD, and GPx in Post-Thaw Sperm

The catalase assay kit (S0051, Beyotime Institute of Biotechnology), total superoxide dismutase assay kit (S0060), and glutathione peroxidase assay kit (S0056) were used to assess the activities of catalase (CAT) and superoxide dismutase (SOD) and GPx, respectively. According to Zhu et al. (2019b) [25], post-thaw sperm pellets were lysed ultrasonically on ice and centrifuged to collect the supernatants. The supernatants were used to analyze the activities of CAT, SOD, and GPx, according to the manufacturer’s instructions.

### 2.11. Annexin V-FITC/PI Assay

Annexin V-FITC/PI apoptosis detection kit (Sigma-Aldrich, St. Louis, MO, USA) was used to assess sperm apoptosis according to the manufacturer’s instruction with slight modifications. The post-thaw sperm was centrifuged and washed with PBS. The post-thaw sperm was centrifuged and washed with PBS. The sperm was resuspended with 1× Annexin V-binding buffer at a concentration of 1 × 10^6^ sperm/mL. A total of 5 μL Annexin V-FITC (AN) and 3 μL PI were then added to each aliquot of 100 μL sample. The tubes were mixed gently and incubated for 10 min in the dark. Different labeling patterns of stained sperm were observed with a fluorescence microscope (80i; Nikon, Melville, NY, USA) with 535 nm excitation and 617 nm emission for red fluorescence (PI) and 488 nm excitation and 516 nm emission for green fluorescence (AN).

### 2.12. Statistical Analysis

Data from three replicates were compared using one-way analysis of variance followed by Tukey’s post hoc test (Statview; Abacus Concepts, Inc., Berkeley, CA, USA). All the values are presented as the mean ± standard error of the mean (SEM). Treatments were considered statistically different from one another at *p* < 0.05.

## 3. Results

### 3.1. Addition of Carboxylated ε-Poly-L-Lysine to the Freezing Medium Increased the Post-Thaw Sperm Motility Parameters

As shown in Table 1, when sperm motility patterns were analyzed by the motility tracks generated from CASA, it was observed that the addition of 0.25% or 0.5% CPLL to the freezing medium significantly increased post-thaw sperm total motility, progressive motility, VSL, VCL, VAP, LIN, and STR. Interestingly, for the aforementioned parameters, the 0.25% CPLL treatment showed the highest value among all treatments. However, there was no significant difference between the treatments in terms of post-thaw sperm ALH and BCF parameters.

### 3.2. Addition of Carboxylated ε-Poly-L-Lysine to the Freezing Medium Improved the Post-Thaw Sperm Membrane Integrity and Acrosome Integrity

**Figure 1:**
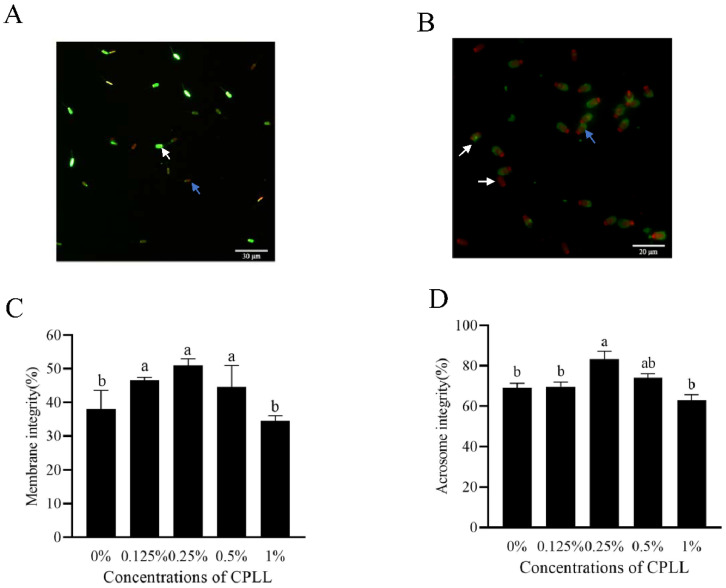
Photos of the post-thaw boar sperm stained with LIVE/DEAD sperm viability kit (**A**) and fluorescein isothiocyanate-peanut agglutinin (**B**) working solution, respectively. In image (**A**), the white arrow presented the post-thaw sperm with membrane integrity while the blue arrow presented post-thaw sperm with damaged integrity. In image (**B**), the white arrow indicated the post-thaw sperm with damaged acrosome, whereas the blue arrow indicated the post-thaw sperm with intact acrosome. Effects of supplementation with different concentrations of CPLL to the freezing medium on post-thaw boar sperm membrane integrity (**C**) and acrosome integrity (**D**). Values are specified as mean ± standard error of the mean (SEM). Columns with different lowercase letters differ significantly (*p* < 0.05), *n* = 5.

In Figure 1A, the white arrow indicated sperm with membrane integrity presented as green fluoresce, meanwhile the blue arrow indicated the sperm with damaged membrane integrity presented as red fluoresce. Moreover, the addition of 0.125% to 0.5% CPLL to the freezing medium significantly increased the post-thaw sperm membrane integrity, and the 0.25% CPLL treatment had the highest value among the treatments (Figure 1C).

In terms of post-thaw sperm acrosome integrity, the blue arrow showed sperm with intensively bright fluorescence in the acrosomal cap were indicated post-thaw sperm with intact acrosome, whereas the white arrow showed sperm with disrupted fluorescence or no fluorescence in the acrosomal cap presented post-thaw sperm with damaged acrosome (Figure 1B). The addition of the CPLL to the freezing medium greatly contributed to maintaining post-thaw sperm intact acrosome (Figure 1D). The 0.25% CPLL treatment showed a significantly higher amount of intact acrosome compared to the control (Figure 1D).

### 3.3. Addition of Carboxylated ε-Poly-L-Lysine to the Freezing Medium Improved the Post-Thaw Sperm Mitochondrial Function

**Figure 2:**
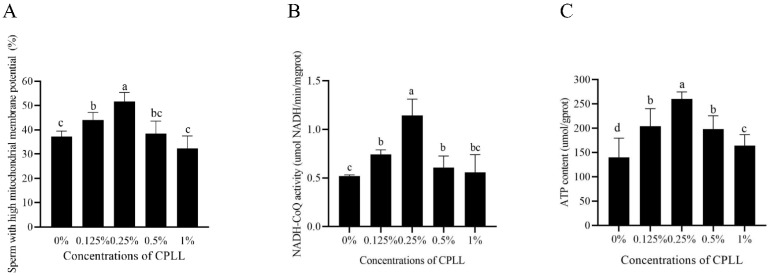
Effects of supplementation with different concentration of CPLL to the freezing medium on post-thaw boar sperm mitochondrial membrane potential (**A**), NADH-CoQ activity (**B**), and ATP level (**C**). Values are specified as mean ± standard error of the mean (SEM). Columns with different lowercase letters differ significantly (*p* < 0.05), *n* = 5.

As shown in Figure 2A, the percentage of post-thaw sperm with high mitochondrial membrane potential was significantly increased by supplementation of CPLL in the freezing medium. Moreover, the maximum value was noted in 0.25% CPLL treatment (Figure 2A). However, the post-thaw sperm mitochondrial membrane potential in 0.5% and 1% CPLL treatments showed no difference compared to the control (Figure 2A).

Interestingly, the values of NADH-CoQ activity in 0.125%, 0.25%, and 0.5% CPLL treatments were significantly higher than the control treatment, and the 0.25% CPLL treatment showed the highest value (Figure 2B). However, the result of NADH-CoQ activity in 1% CPLL treatment was similar to the control (Figure 2B).

### 3.4. Addition of Carboxylated ε-Poly-L-Lysine to the Freezing Medium Increased the Post-Thaw Sperm ATP Level

As shown in Figure 2C, the post-thaw sperm ATP level was significantly increased by supplementation of CPLL (from 0.125% to 1%) to the freezing medium, and the 0.25% CPLL treatment showed the highest ATP level among the treatments.

### 3.5. Addition of Carboxylated ε-Poly-L-Lysine to the Freezing Medium Improved the Post-Thaw Sperm Antioxidative Stress

**Figure 3:**
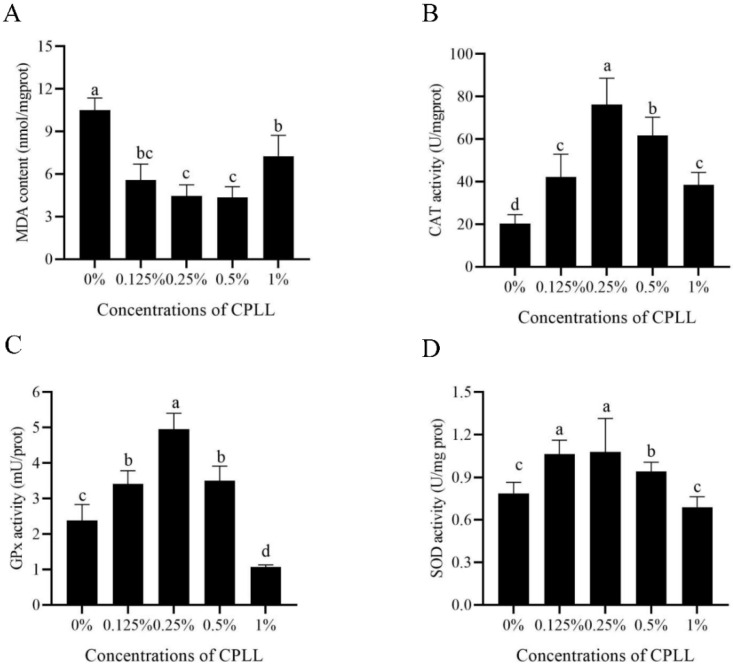
Effects of supplementation with different concentrations of CPLL to the freezing medium on post-thaw boar sperm MDA level (**A**), CAT activity (**B**), GPx activity (**C**), and SOD activity (**D**). Values are specified as mean ± standard error of the mean (SEM). Columns with different lowercase letters differ significantly (*p* < 0.05). CAT, catalase; GPx, glutathione peroxidase; SOD, superoxide dismutase, *n* = 5.

To detect whether CPLL could protect post-thaw boar sperm from oxidative damage, MDA level, activities of CAT, GPx, and SOD in post-thaw sperm were also measured. As shown in Figure 3A, the MDA levels in CPLL treatments were significantly lower than in control. Moreover, 0.25% and 0.5% of CPLL treatments showed the lowest MDA levels. The value of CAT activity was also significantly increased by the addition of CPLL at 0.125% to 1% concentration levels (Figure 3B). Similarly, addition of 0.125%, 0.25%, or 0.5% CPLL to the freezing medium also significantly improved the activities of GPx and SOD; however, a concentration of 1% CPLL treatment could not improve both parameters (Figure 3C,D).

### 3.6. Addition of Carboxylated ε-Poly-L-Lysine to the Freezing Medium Reduced the Post-Thaw Sperm Apoptosis

**Figure 4:**
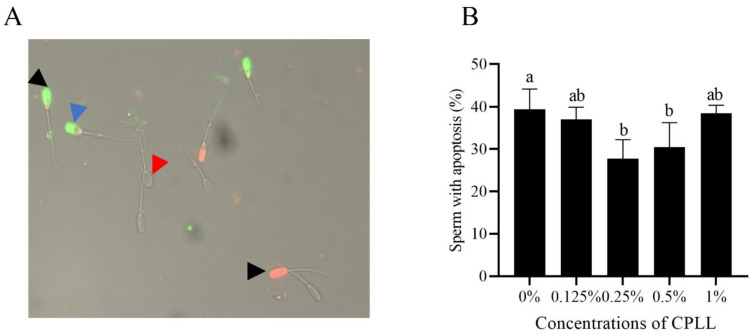
The images of post-thaw sperm stained with the Annexin V-FITC/PI assay kit (**A**). Blue arrow indicates the post-thaw sperm with apoptosis stained with AN^+^/PI^−^ that presented green fluorescence, black arrow indicated the dead sperm stained with AN^+^/PI^+^ or AN^−^/PI^+^ that presented orange or red fluorescence, red arrow indicated the live sperm with no fluorescence. Effects of different concentrations of CPLL on boar post-thaw sperm apoptosis (**B**). Values are specified as mean ± standard error of the mean (SEM). Columns with different uppercase letters differ significantly (*p* < 0 05), *n* = 5.

As shown in Figure 4A, the post-thaw boar sperm stained with AN^+^/PI^−^ (early apoptotic) presented green fluorescence was regarded as the apoptotic sperm (blue arrow), and the necrotic sperm stained with AN^+^/PI^+^ (early necrotic) or AN^−^/PI^+^ (late necrotic) presented orange or red fluorescence (black arrow). Meanwhile, the red arrow indicated the live sperm with no fluorescence. It was observed that addition of 0.25% and 0.5% CPLL significantly decreased the post-thaw sperm apoptosis. However, post-thaw sperm apoptosis in 0.125% and 1% of CPLL treatments were not different compared to the control (Figure 4B).

## 4. Discussion

Cryopreservation is the best tool to preserve structurally intact live sperm for a long period of time in livestock. Although the implementation of AI with frozen sperm has been widely practiced in cattle and horse domestic species, pig farming is a different case due to several practical problems, such as the high cost of the frozen doses and low fertility results and prolificacy after the use of post-thaw boar sperm [3,26]. Generally, in pigs, the mean fertility with liquid storage sperm could reach a percentage above 90%, while fertility with post-thaw boar sperm only reached 80% in the best case. Moreover, AI with post-thaw boar sperm showed 2 to 3 less young born per litter compared to the result of AI using liquid storage sperm [3,27]. It is a concern that AI with post-thaw sperm is not very successful in pigs as it is in cattle [1]. Recently, reproductive researchers and cryobiologists explored freezing–thawing procedures [28], seminal plasma [13,29], pre-treatment medium [14], freezing extenders [3], anti-bacterial, antioxidants [9], and anti-freezing proteins [12] for improving the boar sperm cryopreservation quality. Our present study focused on developing an effective cryoprotectant for boar sperm cryopreservation. It was observed that the addition of 0.25% CPLL to the freezing extender significantly increased the motility, membrane integrity, acrosome integrity, mitochondrial function, and antioxidative capacity of post-thaw sperm.

Cryoprotectants, including nonpermeating and permeating cryoprotectants, are essential chemicals in the freezing medium. The cryoprotectant properties of glycerol were discovered by Polge and colleagues 70 years ago. They found that glycerol penetrates the cell membrane and prevents excessive dehydration during cooling and freezing processes [30]. Glycerol is commonly used at a high concentration of 3–6% (*v/v*) in boar sperm cryopreservation [3,16,26,31]. Several studies were conducted to find new and efficient CAPs to replace or reduce the use of glycerol in boar sperm cryopreservation procedure [32,33]. In the present study, the carboxylated ε-poly-L-lysine (CPLL), which is a polymer, was used as a modern cryoprotectant in boar sperm cryopreservation. The result showed that the addition of 0.25% (*v/v*) CPLL significantly protected the post-thaw sperm function. It is well known that the CPLL with a low concentration can exhibit antifreeze activities by inhibiting ice crystal growth and recrystallization during cryopreservation, which is similar to the properties of anti-freeze proteins [19,34]. Generally, during the freezing–thawing processes, small ice crystals tend to convert to large ice crystals. Because the melting point in small ice crystals is lower than the larger crystals, once they melt, they release liquid water that results in merging with adjacent larger crystals and recrystallize [35]. In addition, the large ice crystals cause sperm damage because of membrane rupture and cell dehydration. Due to the carboxyl group binds to crystals, the ice crystals in CPLL treatment are more hexagonal or bipyramid in shape [19,21,35]. Membrane damage caused by the hexagonal crystal shape is generally considered lower than that in round and flat ice crystals [17], indicating that the CPLL treatment in boar sperm cryopreservation helps to protect sperm membrane integrity. Post-thaw boar sperm membrane integrity and acrosome integrity in the present study were improved by the addition of 0.25% CPLL (*v/v*) (Figure 1). The values of post-thaw boar sperm parameters, such as membrane integrity and acrosome integrity in control, were lower than previous reports of cryopreserved sperm with a normal concentration of glycerol (3%), which might be due to the boar sperm being cryopreserved with low glycerol (1%) in this study. Unfortunately, the effects of different concentrations of glycerol and CPLL cryoprotectant combinations on boar sperm were not investigated; it is a limitation of this study. In addition, during cooling, the CPLL is reported to have high adsorption to the membrane as it can bind to the membrane in a similar way to the anti-freeze proteins for protection of the membrane from outside and to maintain membrane integrity [19,36]. Moreover, the CPLL not only binds to the membrane but also has a high affinity to water, suggesting that CPLL can also protect the sperm membrane from cryo-damage by removing the sperm intracellular water to reduce crystal formation and growth. Therefore, the addition of CPLL to the freezing medium helps improve not only the post-thaw boar sperm membrane integrity but also acrosome integrity.

Sperm motility is a predicted parameter of fertilization as the sperm need to move from the ejaculated or deposited sites to the oviduct where it penetrates the oocytes in vivo [37]. In the present study, when sperm motility patterns were analyzed with CASA, it was observed that the total motility, progressive motility, VSL, VCL, VAP, STR, and LIN patterns of post-thaw sperm in 0.25% CPLL treatment were much higher than the non-CPLL treatment, indicating that addition of CPLL to the freezing medium contributed to the post-thaw boar sperm motility. The result was supported by Fujikawa et al. (2018), who reported that supplementation of CPLL increased the bovine sperm motility [38]. Sperm motility has a positive correlation with membrane integrity and acrosome integrity. Thus, the increase in post-thaw sperm motility may be due to the protection of boar sperm membrane from cryo-damage by the addition of CPLL in the present study.

Keeping sperm mitochondrial function is important for maintaining sperm function. Damage of sperm mitochondrial enzymes [39], mitochondrial DNA integrity [40], or mitochondrial membrane potential [41] had been observed with a reduction of sperm fertility. A previous study reported that a peak in oxygen consumption was observed when the boar sperm induced capacitation in vitro [42], suggesting that the mitochondrial function is required for sperm capacitation. In addition, maintenance of boar sperm motility was also needed to keep sperm mitochondrial function as the mitochondrial central dogma was crucial to sperm motility [43]. However, the freezing–thawing processes damaged sperm mitochondrial function [9,44]. Therefore, strategies for improving the post-thaw boar sperm mitochondrial function would enhance the cryopreserved boar sperm utilization. In the present study, it was found that the addition of 0.25% CPLL could increase the post-thaw boar sperm mitochondrial membrane potential and NADH-CoQ activity for generating ATP to improve the post-thaw sperm quality. Those data were supported by Tariq et al. (2020), who found that supplementation of CPLL increased the value of Nili Ravi buffalo bull sperm with high mitochondrial membrane potential [23]. In addition, the ATP level observed with an increase in 0.25% CPLL treatment consisted of the fact in which sperm ATP generated from glycolysis and oxidative phosphorylation pathway [45] as the CPLL protected the sperm mitochondrial function. From those observations, the addition of CPLL to the freezing medium will be beneficial for improving the post-thaw boar quality by protecting the mitochondrial function.

Notably, boar sperm contains endogenous enzymatic antioxidants which act as free radical scavengers to protect them against oxidative damage during the freezing and thawing process [9,46]. The enzymatic antioxidants in sperm are important for keeping their quality by reducing oxidative damage [46]. It is also well known that boar sperm is rich in polyunsaturated fatty acids (PUFAs) that are susceptible to cryo-damage [24]. The PUFAs in sperm membrane undergo lipid peroxidation to induce apoptosis via disrupting the membrane fluidity when the sperm antioxidant defense system was impaired during the freezing and thawing processes [9,47]. Therefore, during the cryopreservation, maintaining the boar sperm antioxidative ability is important. In this study, a higher value of activities of CAT, SOD, and GPx in 0.25% CPLL treatment was observed in post-thaw boar sperm, indicating that post-thaw boar sperm antioxidative stress was improved by adding CPLL. In fact, those data were consistent with the reduction of MDA level and supported by a previous report that supplementation of CPLL decreased the MDA level in Nili Ravi buffalo bull sperm [23]. Generally, when the sperm membrane was damaged by ROS, the membrane permeability was irreversibly interrupted, thereby inducing the mitochondria to release cytochrome C for activation of caspase-9 and caspase-3 to promote apoptosis [48,49]. In this study, the reduction of boar sperm with apoptosis in CPLL treatment was observed, which may be due to CPLL decreasing the ROS damage via enhancement of the sperm antioxidative ability. Those observations were consistent with Zhu et al. (2019) that reduction of ROS damage decreased the apoptosis during the boar sperm cryopreservation [9]. Therefore, from those results, CPLL reduced the sperm oxidative stress for decreasing apoptosis via protecting the post-thaw sperm antioxidant defense system.

## 5. Conclusions

In conclusion, the present study showed that addition of CPLL to the freezing medium improved the post-thaw boar sperm motility, membrane integrity, acrosome integrity, and ATP level, along with an increase in mitochondrial function and antioxidant defense system; meanwhile, it decreased MDA level and apoptosis of post-thaw sperm. Those observations indicated that CPLL can be used as an effective cryoprotectant in boar sperm cryopreservation.

## Figures and Tables

**Table 1:** Effect of CPLL on post-thaw boar sperm motility parameters measured with CASA.

Sperm Parameters	0%	0.125%	0.25%	0.5%	1%
Total motility (%)	36.2 ± 3.6 ^b^	34.4 ± 4.0 ^b^	58.2 ± 5.0 ^a^	53.2 ± 2.1 ^a^	41.5 ± 5.3 ^ab^
Progressive motility (%)	17.7 ± 1.2 ^c^	19.2 ± 2.7 ^c^	43.0 ± 4.3 ^a^	32.8 ± 3.2 ^b^	23.3 ± 3.5 ^c^
VCL (μm/s)	117.4 ± 9.1 ^b^	115.4 ± 4.6 ^b^	144.4 ± 4.4 ^a^	139.3± 3.0 ^a^	125.3 ± 8.0 ^b^
VSL (μm/s)	32.6 ± 3.3 ^b^	28.5 ± 1.8 ^b^	48.7± 3.0 ^a^	50.1 ± 2.3 ^a^	37.9 ± 5.1 b
VAP (μm/s)	61.2 ± 4.5 ^b^	64.3 ± 3.8 ^b^	75.1 ± 2.1 ^a^	74.0 ± 0.8 ^a^	67.8 ± 4.6 ^ab^
BCF (Hz)	24.7 ± 1.2	24.5 ± 1.4	26.4 ± 0.9	24.9 ± 0.6	24.2 ± 1.2
ALH (μm)	8.7 ± 0.6	8.9 ± 0.3	8.3 ± 0.4	8.2 ± 0.3	8.4 ± 0.3
STR (%)	47.5 ± 2.0 ^b^	44.8 ± 1.3 ^b^	63.6 ± 3.3 ^a^	66.4 ± 2.6 ^a^	54.2 ± 4.2 ^ab^
LIN (%)	26.1 ± 1.0 ^b^	25.1 ± 1.0 ^b^	33.8 ± 2.1 ^a^	36.1 ± 1.9 ^a^	29.8 ± 2.1 ^ab^

(Values are expressed as mean ± standard deviation. Different letters within column the same row indicate significant difference (*p* < 0.05). VCL, curvilinear velocity; VSL, straight-line velocity; VAP, average path velocity; BCF, beat-cross frequency; ALH, lateral head; STR, straightness (VSL/VAP); LIN, linearity (VSL/VCL); WOB, wobble (VAP/VCL); *n* = 5).

## Data Availability

The data presented in this study are available in the article.
